# New Insights into Impaired Muscle Glycogen Synthesis: More a Problem of Muscle Contractility Than of Glucose Tolerance

**DOI:** 10.1371/journal.pmed.0050025

**Published:** 2008-01-29

**Authors:** Leif Groop, Marju Orho-Melander

## Abstract

The authors discuss a new study showing that carriers of the mutation PPP1R3A show decreased muscle glycogen levels.

Glucose is the most rapidly accessible substrate in the body. Its storage as glycogen in muscle and liver is of central importance as a first source of energy for muscle contractions and prevention against hypoglycemia. Glycogen synthesis and breakdown are regulated by insulin and catecholamines as well as by glucose-6-phosphate and the amount of glycogen. There are two isoforms of glycogen synthase (GS), one in muscle and one in liver, encoded by different genes (*GYS1* and *GYS2*, respectively) [1]. Insulin stimulates glycogen synthesis by activating protein phosphatase 1 (PP1), which activates GS, inactivates glycogen phosphatase, and inactivates glycogen synthase kinase 3, an inhibitor of GS. PP1 has a glycogen-targeting subunit (PPP1R3A), which facilitates localization of PP1 to glycogen.

## Impaired Insulin-Stimulated Glycogen Synthesis Precedes Type 2 Diabetes

It is well established that patients with type 2 diabetes as well as persons at risk of the disease, i.e., first-degree relatives of patients with type 2 diabetes, are insulin resistant and show impaired insulin-stimulated glycogen synthesis [2,3], possibly as a consequence of impaired stimulation of GS by insulin [4]. It has therefore been suggested that impaired glycogen synthesis could be central in the pathogenesis of type 2 diabetes. A common variant in the *GYS1* gene has been associated with type 2 diabetes and insulin resistance, as well as increased risk of cardiovascular morbidity and mortality [5,6]. Carriers of the variant allele of this polymorphism are not able to increase their GS protein in response to exercise [7]. Interestingly, there seems to be an interaction between this polymorphism and exercise in the prevention of cardiovascular disease, i.e., risk genotype carriers do not experience the protective effect of exercise on cardiovascular risk [8].

This Perspective discusses the following new study published in *PLoS Medicine*:

Savage DB, Zhai L, Ravikumar B, Choi CS, Snaar JE, et al. (2008) A prevalent variant in *PPP1R3A* impairs glycogen synthesis and reduces muscle glycogen content in humans and mice. PLoS Med 5: e27. doi:10.1371/journal.pmed.0050027

Stephen O'rahilly and colleagues describe the effect of a mutation in *PPP1R3A*, present in 1.36% of participants from one UK population, that directly impairs glycogen synthesis and decreases glycogen levels in human skeletal muscle.

## Inborn Errors of Glycogen Synthesis

Although several inborn errors of metabolism affect breakdown of glycogen (leading to glycogen storage diseases), until recently no genetic defects have been known to affect glycogen synthesis in muscle, only in liver [9]. Newborn children with mutations in both alleles of their *GYS2* gene present with severe hypoglycemia. In 2002 a digenic form of severe insulin resistance was reported: in a human pedigree, the patients carried both a stop/frameshift mutation in the regulatory subunit of *PPP1R3A* as well as a mutation in the gene encoding for the transcription factor PPAR [10]. Now, in a new study published in *PLoS Medicine*, the same authors demonstrate that 1.4% (one out of 70) of whites in the United Kingdom carry this stop/frameshift mutation in *PPP1R3A*, and that carriers of this mutation show decreased muscle glycogen levels as measured by ^13^C mass spectroscopy [11]. They also developed a mouse model carrying the human mutation. In muscle tissue from these mice, the mutant protein failed to bind to glycogen, thus decreasing GS activity and glycogen synthesis. However, there was no increased frequency of diabetes in mutation carriers in the mice.

The authors claim that this is the first genetic defect shown to specifically decrease skeletal muscle glycogen synthesis and content. This was certainly true when this work was performed, but very recently a homozygous mutation was described in the *GYS1* gene in children with severe cardiomyopathy and exercise intolerance [12]. In accordance with the liver disease caused by mutations in the *GYS2* gene, the disease was called muscle glycogen storage disease 0. The number 0 here implies that there is little or no glycogen formed.

## What Are the Clinical Implications?

There are several important implications of these two papers [11,12]. First, genetic defects *do* cause impaired glycogen synthesis in muscle. Second, impaired glycogen synthesis in muscle does not a priori lead to insulin resistance, impaired glucose tolerance, or diabetes, since glucose tolerance was normal in carriers of mutations in both the *PPP1R3A* and *GYS1* genes. By analogy, glucose tolerance was also normal in mice carrying a mutation in the muscle glycogen synthase gene (the *MGSKO* mouse) [13]. Rather than affecting glucose tolerance, glycogen deficiency in muscle seems to influence exercise and cardiac performance. It seems that heart muscle, which has twice as much glycogen as skeletal muscle, is more vulnerable to disturbances in glycogen metabolism than skeletal muscle, since glycogen breakdown is the fastest source of energy in the heart. It would be important to study if carriers of the *PPP1R3A* variant show differences in their exercise and cardiac performance.

Given that mutations in *PPP1R3A* are seen in one out of 70, and in *GYS1* in one out of 100 white people, it will be important to screen for these mutations in individuals with signs of exercise intolerance or cardiomyopathy.

Although impaired glycogen synthesis is a hallmark of insulin resistance and precedes type 2 diabetes, it seems unlikely that it is involved in the pathogenesis of the disease. Impaired glycogen synthesis is rather the consequence of insulin resistance in more proximal steps of intracellular glucose metabolism. In contrast, the previous findings of increased risk of cardiovascular morbidity and mortality in carriers of a common variant in the *GYS1* gene may point to the central role of glycogen metabolism for heart muscle.

Taken together, these studies have shed new light on the role of disturbed glycogen synthesis in disease pathogenesis. Genetic screening can now be combined with relevant cardiorespiratory exercise tests and muscle biopsies in individuals with unclear symptoms of exercise fatigue and poor cardiac performance.

## Abbreviations

GS: glycogen synthase
PP1: protein phosphatase 1
